# Supplementary figures and images for: Identification of key genes and long non-coding RNA associated ceRNA networks in hepatocellular carcinoma
Source: PeerJ. 2019 Nov 1;7:e8021. doi: 10.7717/peerj.8021 (PMC6827457; doi:10.7717/peerj.8021)

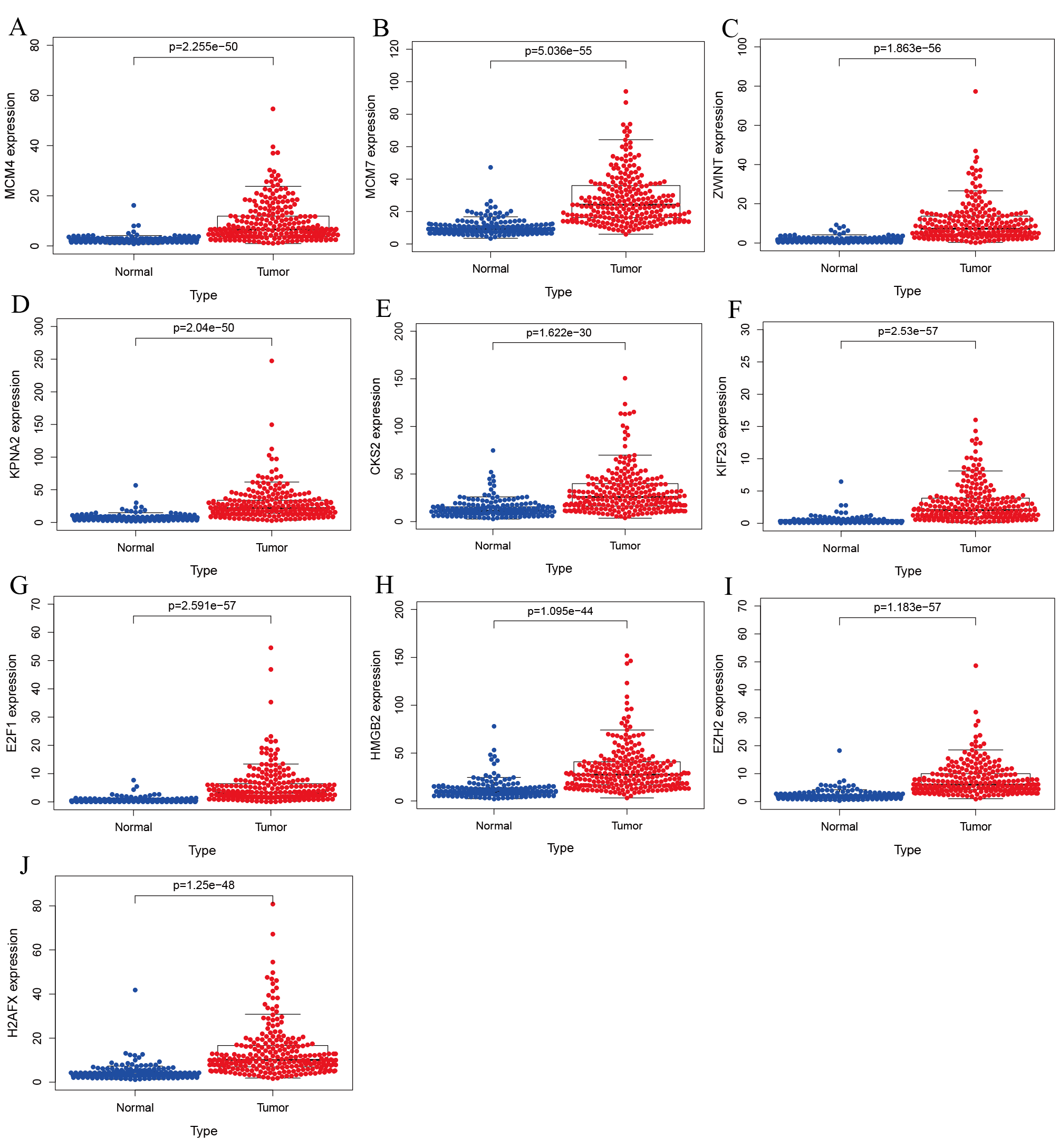

Supplement: Supplemental Information 3 — (A) MCM4. (B) MCM7. (C) ZWINT. (D) KPNA2. (E) CKS2. (F) KIF23. (G) E2F1. (H) HMGB2. (I) EZH2. (J) H2AFX. All hub genes were significantly up-regulated in tumor tissues than in normal ones. [file peerj-07-8021-s003.png]

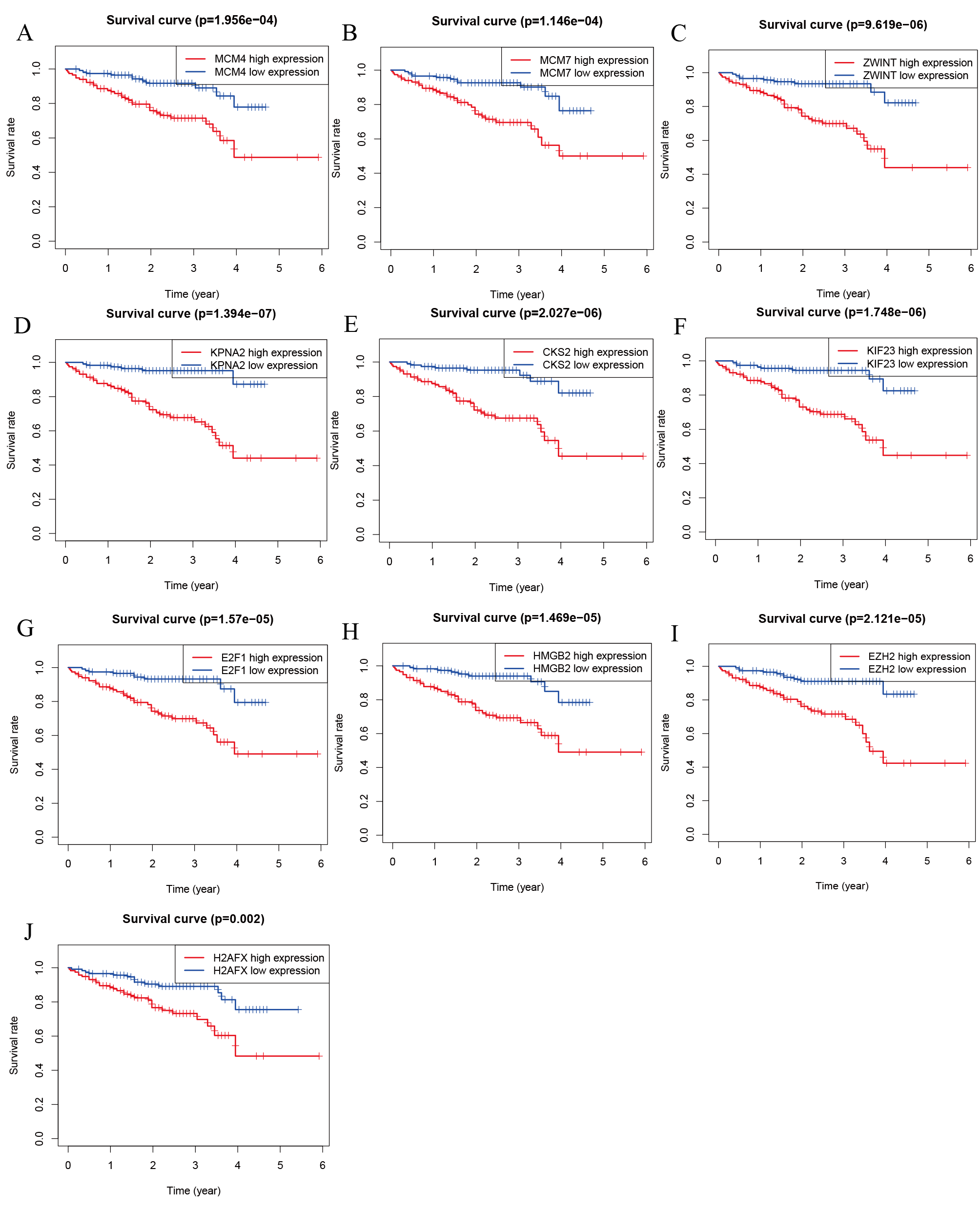

Supplement: Supplemental Information 4 — (A) MCM4. (B) MCM7. (C) ZWINT. (D) KPNA2. (E) CKS2. (F) KIF23. (G) E2F1. (H) HMGB2. (I) EZH2. (J) H2AFX. HCC patients with high expression of any of the hub genes had shorter overall survival time. [file peerj-07-8021-s004.png]
