# Supplementary material for: Identification of key genes and long non-coding RNA associated ceRNA networks in hepatocellular carcinoma
Source: PeerJ. 2019 Nov 1;7:e8021. doi: 10.7717/peerj.8021 (PMC6827457; doi:10.7717/peerj.8021)
Supplement: Supplemental Information 5 [file peerj-07-8021-s005.docx]

**Table S1** Target miRNAs of DElncRNAs by prediction

| lncRNA | miRNA |
| --- | --- |
| FAM182B | hsa-miR-551a, hsa-miR-143, hsa-miR-1721, hsa-miR-4770, hsa-miR-144, hsa-miR-146ac, hsa-miR-146b-5p, hsa-miR-193, hsa-miR-193b, hsa-miR-193a-3p, hsa-miR-199ab-5p, hsa-miR-1ab, hsa-miR-206, hsa-miR-613, hsa-miR-203, hsa-miR-218, hsa-miR-218a, hsa-miR-25, hsa-miR-32, hsa-miR-92abc, hsa-miR-363, hsa-miR-363-3p, hsa-miR-367, hsa-miR-29abcd, hsa-miR-103a, hsa-miR-107, hsa-miR-107ab, hsa-miR-34ac, hsa-miR-34bc-5p, hsa-miR-449abc, hsa-miR-449c-5p, hsa-miR-125a-5p, hsa-miR-125b-5p, hsa-miR-351, hsa-miR-670, hsa-miR-4319, hsa-miR-129-5p, hsa-miR-129ab-5p |
| FAM138D | hsa-miR-135ab, hsa-miR-135a-5p, hsa-miR-150, hsa-miR-5127, hsa-miR-15abc, hsa-miR-16, hsa-miR-16abc, hsa-miR-195, hsa-miR-322, hsa-miR-424, hsa-miR-497, hsa-miR-1907, hsa-miR-18ab, hsa-miR-4735-3p, hsa-miR-192, hsa-miR-215, hsa-miR-200bc, hsa-miR-429, hsa-miR-548a, hsa-miR-204, hsa-miR-204b, hsa-miR-211, hsa-miR-214, hsa-miR-761, hsa-miR-3619-5p, hsa-miR-216a, hsa-miR-216b, hsa-miR-216b-5p, hsa-miR-24, hsa-miR-24ab, hsa-miR-24-3p, hsa-miR-27abc, hsa-miR-27a-3p, hsa-miR-103a, hsa-miR-107, hsa-miR-107ab, hsa-miR-383 |
| UCA1 | hsa-miR-96, hsa-miR-507, hsa-miR-1271, hsa-miR-135ab, hsa-miR-135a-5p, hsa-miR-138, hsa-miR-138ab, hsa-miR-143, hsa-miR-1721, hsa-miR-4770, hsa-miR-182, hsa-miR-184, hsa-miR-18ab, hsa-miR-4735-3p, hsa-miR-190, hsa-miR-190ab, hsa-miR-193, hsa-miR-193b, hsa-miR-193a-3p, hsa-miR-1ab, hsa-miR-206, hsa-miR-613, hsa-miR-203, hsa-miR-214, hsa-miR-761, hsa-miR-3619-5p, hsa-miR-122, hsa-miR-122a, hsa-miR-1352, hsa-miR-23abc, hsa-miR-23b-3p, hsa-miR-26ab, hsa-miR-1297, hsa-miR-4465, hsa-miR-103a, hsa-miR-107, hsa-miR-107ab, hsa-miR-124, hsa-miR-124ab, hsa-miR-506, hsa-miR-383, hsa-miR-455-5p, hsa-miR-129-5p, hsa-miR-129ab-5p |
| FAM138C | hsa-miR-150, hsa-miR-5127, hsa-miR-15abc, hsa-miR-16, hsa-miR-16abc, hsa-miR-195, hsa-miR-322, hsa-miR-424, hsa-miR-497, hsa-miR-1907, hsa-miR-200bc, hsa-miR-429, hsa-miR-548a, hsa-miR-204, hsa-miR-204b, hsa-miR-211, hsa-miR-214, hsa-miR-761, hsa-miR-3619-5p, hsa-miR-216a, hsa-miR-216b, hsa-miR-216b-5p, hsa-miR-24, hsa-miR-24ab, hsa-miR-24-3p, hsa-miR-27abc, hsa-miR-27a-3p, hsa-miR-103a, hsa-miR-107, hsa-miR-107ab, hsa-miR-383 |
| FAM138B | hsa-miR-9, hsa-miR-9ab, hsa-miR-150, hsa-miR-5127, hsa-miR-15abc, hsa-miR-16, hsa-miR-16abc, hsa-miR-195, hsa-miR-322, hsa-miR-424, hsa-miR-497, hsa-miR-1907, hsa-miR-18ab, hsa-miR-4735-3p, hsa-miR-200bc, hsa-miR-429, hsa-miR-548a, hsa-miR-204, hsa-miR-204b, hsa-miR-211, hsa-miR-214, hsa-miR-761, hsa-miR-3619-5p, hsa-miR-216a, hsa-miR-216b, hsa-miR-216b-5p, hsa-miR-122, hsa-miR-122a, hsa-miR-1352, hsa-miR-24, hsa-miR-24ab, hsa-miR-24-3p, hsa-miR-27abc, hsa-miR-27a-3p, hsa-miR-103a, hsa-miR-107, hsa-miR-107ab, hsa-miR-383 |
| FAM41C | hsa-miR-503, hsa-miR-7, hsa-miR-7ab, hsa-miR-139-5p, hsa-miR-141, hsa-miR-200a, hsa-miR-144, hsa-miR-145, hsa-miR-153, hsa-miR-183, hsa-miR-187, hsa-miR-18ab, hsa-miR-4735-3p, hsa-miR-199ab-5p, hsa-miR-19ab, hsa-miR-1ab, hsa-miR-206, hsa-miR-613, hsa-miR-205, hsa-miR-205ab, hsa-miR-27abc, hsa-miR-27a-3p, hsa-miR-101, hsa-miR-101ab, hsa-miR-31, hsa-miR-124, hsa-miR-124ab, hsa-miR-506, hsa-miR-125a-5p, hsa-miR-125b-5p, hsa-miR-351, hsa-miR-670, hsa-miR-4319, hsa-miR-455-5p |
| GAS5 | hsa-miR-132, hsa-miR-212, hsa-miR-212-3p, hsa-miR-93, hsa-miR-93a, hsa-miR-105, hsa-miR-106a, hsa-miR-291a-3p, hsa-miR-294, hsa-miR-295, hsa-miR-302abcde, hsa-miR-372, hsa-miR-373, hsa-miR-428, hsa-miR-519a, hsa-miR-520be, hsa-miR-520acd-3p, hsa-miR-1378, hsa-miR-1420ac, hsa-miR-96, hsa-miR-507, hsa-miR-1271, hsa-miR-135ab, hsa-miR-135a-5p, hsa-miR-137, hsa-miR-137ab, hsa-miR-138, hsa-miR-138ab, hsa-miR-139-5p, hsa-miR-144, hsa-miR-148ab-3p, hsa-miR-152, hsa-miR-153, hsa-miR-155, hsa-miR-182, hsa-miR-18ab, hsa-miR-4735-3p, hsa-miR-196abc, hsa-miR-200bc, hsa-miR-429, hsa-miR-548a, hsa-miR-205, hsa-miR-205ab, hsa-miR-208ab, hsa-miR-208ab-3p, hsa-miR-21, hsa-miR-590-5p, hsa-miR-216a, hsa-miR-216b, hsa-miR-216b-5p, hsa-miR-217, hsa-miR-221, hsa-miR-222, hsa-miR-222ab, hsa-miR-1928, hsa-miR-223, hsa-miR-23abc, hsa-miR-23b-3p, hsa-miR-24, hsa-miR-24ab, hsa-miR-24-3p, hsa-miR-26ab, hsa-miR-1297, hsa-miR-4465, hsa-miR-29abcd, hsa-miR-31, hsa-miR-33a-3p, hsa-miR-365, hsa-miR-365-3p, hsa-miR-425, hsa-miR-425-5p, hsa-miR-489, hsa-miR-10abc, hsa-miR-10a-5p, hsa-miR-455-5p, hsa-miR-128, hsa-miR-128ab, hsa-miR-490-3p, hsa-miR-499-5p |
| SNHG3 | hsa-miR-93, hsa-miR-93a, hsa-miR-105, hsa-miR-106a, hsa-miR-291a-3p, hsa-miR-294, hsa-miR-295, hsa-miR-302abcde, hsa-miR-372, hsa-miR-373, hsa-miR-428, hsa-miR-519a, hsa-miR-520be, hsa-miR-520acd-3p, hsa-miR-1378, hsa-miR-1420ac, hsa-miR-135ab, hsa-miR-135a-5p, hsa-miR-139-5p, hsa-miR-141, hsa-miR-200a, hsa-miR-146ac, hsa-miR-146b-5p, hsa-miR-148ab-3p, hsa-miR-152, hsa-miR-17, hsa-miR-17-5p, hsa-miR-20ab, hsa-miR-20b-5p, hsa-miR-106ab, hsa-miR-427, hsa-miR-518a-3p, hsa-miR-519d, hsa-miR-182, hsa-miR-196abc, hsa-miR-19ab, hsa-miR-1ab, hsa-miR-206, hsa-miR-613, hsa-miR-203, hsa-miR-205, hsa-miR-205ab, hsa-miR-208ab, hsa-miR-208ab-3p, hsa-miR-214, hsa-miR-761, hsa-miR-3619-5p, hsa-miR-216a, hsa-miR-219-5p, hsa-miR-508, hsa-miR-508-3p, hsa-miR-4782-3p, hsa-miR-221, hsa-miR-222, hsa-miR-222ab, hsa-miR-1928, hsa-miR-122, hsa-miR-122a, hsa-miR-1352, hsa-miR-24, hsa-miR-24ab, hsa-miR-24-3p, hsa-miR-101, hsa-miR-101ab, hsa-miR-31, hsa-miR-338, hsa-miR-338-3p, hsa-miR-10abc, hsa-miR-10a-5p, hsa-miR-455-5p, hsa-miR-128, hsa-miR-128ab, hsa-miR-129-5p, hsa-miR-129ab-5p, hsa-miR-490-3p, hsa-miR-499-5p |
| SNHG6 | hsa-miR-7, hsa-miR-7ab, hsa-miR-135ab, hsa-miR-135a-5p, hsa-miR-137, hsa-miR-137ab, hsa-miR-139-5p, hsa-miR-144, hsa-miR-146ac, hsa-miR-146b-5p, hsa-miR-181abcd, hsa-miR-4262, hsa-miR-let-7, hsa-miR-98, hsa-miR-4458, hsa-miR-4500, hsa-miR-193, hsa-miR-193b, hsa-miR-193a-3p, hsa-miR-200bc, hsa-miR-429, hsa-miR-548a, hsa-miR-203, hsa-miR-204, hsa-miR-204b, hsa-miR-211, hsa-miR-205, hsa-miR-205ab, hsa-miR-208ab, hsa-miR-208ab-3p, hsa-miR-214, hsa-miR-761, hsa-miR-3619-5p, hsa-miR-22, hsa-miR-22-3p, hsa-miR-223, hsa-miR-24, hsa-miR-24ab, hsa-miR-24-3p, hsa-miR-26ab, hsa-miR-1297, hsa-miR-4465, hsa-miR-27abc, hsa-miR-27a-3p, hsa-miR-101, hsa-miR-101ab, hsa-miR-30abcdef, hsa-miR-30abe-5p, hsa-miR-384-5p, hsa-miR-34ac, hsa-miR-34bc-5p, hsa-miR-449abc, hsa-miR-449c-5p, hsa-miR-490-3p, hsa-miR-499-5p |
| FAM138E | hsa-miR-150, hsa-miR-5127, hsa-miR-15abc, hsa-miR-16, hsa-miR-16abc, hsa-miR-195, hsa-miR-322, hsa-miR-424, hsa-miR-497, hsa-miR-1907, hsa-miR-200bc, hsa-miR-429, hsa-miR-548a, hsa-miR-204, hsa-miR-204b, hsa-miR-211, hsa-miR-214, hsa-miR-761, hsa-miR-3619-5p, hsa-miR-216a, hsa-miR-216b, hsa-miR-216b-5p, hsa-miR-24, hsa-miR-24ab, hsa-miR-24-3p, hsa-miR-27abc, hsa-miR-27a-3p, hsa-miR-103a, hsa-miR-107, hsa-miR-107ab |
| SCARNA9 | hsa-miR-210, hsa-miR-216a |
| SNHG1 | hsa-miR-503, hsa-miR-7, hsa-miR-7ab, hsa-miR-9, hsa-miR-9ab, hsa-miR-137, hsa-miR-137ab, hsa-miR-140, hsa-miR-140-5p, hsa-miR-876-3p, hsa-miR-1244, hsa-miR-141, hsa-miR-200a, hsa-miR-143, hsa-miR-1721, hsa-miR-4770, hsa-miR-144, hsa-miR-145, hsa-miR-146ac, hsa-miR-146b-5p, hsa-miR-153, hsa-miR-15abc, hsa-miR-16, hsa-miR-16abc, hsa-miR-195, hsa-miR-322, hsa-miR-424, hsa-miR-497, hsa-miR-1907, hsa-miR-181abcd, hsa-miR-4262, hsa-miR-182, hsa-miR-18ab, hsa-miR-4735-3p, hsa-miR-194, hsa-miR-199ab-5p, hsa-miR-1ab, hsa-miR-206, hsa-miR-613, hsa-miR-204, hsa-miR-204b, hsa-miR-211, hsa-miR-205, hsa-miR-205ab, hsa-miR-208ab, hsa-miR-208ab-3p, hsa-miR-21, hsa-miR-590-5p, hsa-miR-216b, hsa-miR-216b-5p, hsa-miR-217, hsa-miR-122, hsa-miR-122a, hsa-miR-1352, hsa-miR-23abc, hsa-miR-23b-3p, hsa-miR-25, hsa-miR-32, hsa-miR-92abc, hsa-miR-363, hsa-miR-363-3p, hsa-miR-367, hsa-miR-101, hsa-miR-101ab, hsa-miR-124, hsa-miR-124ab, hsa-miR-506, hsa-miR-383, hsa-miR-128, hsa-miR-128ab, hsa-miR-129-5p, hsa-miR-129ab-5p, hsa-miR-499-5p |

**Table S2** 126 mRNAs from the first lncRNA-associated ceRNA network

| mRNA |  | |
| --- | --- | --- |
| PEA15 | PEG10 | C7orf43 |
| SOX4 | SLC7A1 | ZFP36L1 |
| SMOC1 | TPD52L1 | KAT2B |
| ABL2 | SCAMP5 | VCAN |
| KDM1A | SQSTM1 | HMGB2 |
| MSMO1 | CA2 | MT1E |
| SORBS2 | TIAM1 | KLHDC10 |
| ITCH | SEMA6D | ZWINT |
| LRRC59 | LAMC1 | SMARCD1 |
| MAGI1 | BCL7A | AHNAK2 |
| NFE2L2 | PTGFRN | PRRG4 |
| KIAA1109 | GPD2 | YWHAH |
| ZBTB9 | FBLIM1 | CDH5 |
| DDX55 | RGPD4 | LPCAT1 |
| UHMK1 | LIFR | MYO1B |
| S1PR1 | MAP1B | TSPAN12 |
| SDHD | LOXL2 | MCM7 |
| FAM129A | DUSP10 | PLAU |
| YWHAZ | PTP4A1 |  |
| G6PD | MCM4 |  |
| UBE2Q2 | IRAK1 |  |
| FAM57A | C1S |  |
| ZNF532 | HIC2 |  |
| ABCC5 | KPNA2 |  |
| PLXNA1 | USP3 |  |
| PIK3R1 | RAP2A |  |
| SGTB | CHAF1A |  |
| UBE2Z | TULP4 |  |
| KLF10 | H2AFX |  |
| ZBTB7A | MARCKSL1 |  |
| ACSL4 | TRIM37 |  |
| RBL2 | SACS |  |
| RREB1 | ARID3B |  |
| INSIG1 | MTSS1 |  |
| TKT | BCL2L11 |  |
| ABHD2 | EIF5 |  |
| E2F1 | APC |  |
| EZH2 | MMD |  |
| TUBB2A | FOS |  |
| PFKP | SLC7A11 |  |
| TOB1 | CAMSAP2 |  |
| HSPA13 | PSD3 |  |
| FJX1 | TNFRSF21 |  |
| RAPGEF4 | SLC1A5 |  |
| MAP3K9 | RBPMS2 |  |
| PPP1R3B | CKS2 |  |
| KIF23 | GPAM |  |
| MRPS23 | CSE1L |  |
| STX6 | OTUD7B |  |
| RNF165 | GOLGA3 |  |
| SIK1 | AVL9 |  |
| BAMBI | MCC |  |
| PRDM1 | IL6R |  |
| CADM1 | CPEB3 |  |

| RNA type | Gene | P-value | Type | Minimum | Lower quartile | Median | Upper quartile | Maximum |
| --- | --- | --- | --- | --- | --- | --- | --- | --- |
| LncRNA | FAM182B | 1.63E-12 | Normal | 0 | 0.022 | 0.043 | 0.057 | 0.099 |
|  |  |  | HCC | 0 | 0.058 | 0.125 | 0.247 | 0.702 |
|  | SNHG1 | 1.62E-12 | Normal | 1.182 | 3.021 | 4.083 | 5.314 | 9.626 |
|  |  |  | HCC | 2.843 | 12.511 | 17.735 | 27.701 | 58.453 |
|  | SNHG3 | 1.62E-12 | Normal | 0.018 | 0.166 | 0.26 | 0.439 | 0.828 |
|  |  |  | HCC | 0.12 | 0.626 | 1.025 | 1.677 | 4.477 |
|  | SNHG6 | <1E-12 | Normal | 20.788 | 42.517 | 54.643 | 66.041 | 86.629 |
|  |  |  | HCC | 35.129 | 82.297 | 127.172 | 212.599 | 463.41 |
| mRNA | MCM4 | 1.62E-12 | Normal | 0.457 | 1.628 | 2.443 | 3.052 | 5.105 |
|  |  |  | HCC | 0.35 | 4.431 | 8.259 | 16.251 | 40.282 |
|  | MCM7 | 1.11E-16 | Normal | 4.38 | 7.007 | 8.631 | 10.68 | 15.828 |
|  |  |  | HCC | 4.471 | 18.941 | 30.707 | 45.311 | 96.717 |
|  | ZWINT | <1E-12 | Normal | 0.234 | 0.62 | 0.869 | 1.532 | 2.638 |
|  |  |  | HCC | 0.739 | 4.836 | 9.789 | 18.766 | 44.58 |
|  | KPNA2 | 1.62E-12 | Normal | 2.383 | 5.952 | 9.105 | 12.682 | 22.337 |
|  |  |  | HCC | 4.172 | 20.507 | 31.836 | 53.627 | 114.624 |
|  | CKS2 | 1.62E-12 | Normal | 5.175 | 7.936 | 11.354 | 14.471 | 23.437 |
|  |  |  | HCC | 6.099 | 23.399 | 35.974 | 61.236 | 137.609 |
|  | KIF23 | 1.62E-12 | Normal | 0.009 | 0.054 | 0.085 | 0.144 | 0.252 |
|  |  |  | HCC | 0.018 | 0.56 | 1.384 | 3.246 | 9.602 |
|  | E2F1 | 1.62E-12 | Normal | 0.031 | 0.155 | 0.225 | 0.313 | 0.708 |
|  |  |  | HCC | 0.233 | 2.731 | 5.96 | 12.572 | 31.233 |
|  | HMGB2 | 1.62E-12 | Normal | 3.98 | 7.465 | 9.622 | 12.009 | 17.281 |
|  |  |  | HCC | 4.786 | 19.612 | 31.021 | 48.311 | 114.746 |
|  | EZH2 | <1E-12 | Normal | 0.062 | 0.322 | 0.428 | 0.71 | 1.426 |
|  |  |  | HCC | 0.263 | 2.245 | 4.088 | 6.944 | 17.069 |
|  | H2AFX | <1E-12 | Normal | 2.467 | 4.392 | 5.384 | 7.036 | 11.013 |
|  |  |  | HCC | 2.584 | 11.599 | 19.077 | 32.597 | 70.699 |

**Table S3** Expression validation of hub genes and DElncRNAs from UALCAN database
